# Proteoglycan-4 predicts good prognosis in patients with hepatocellular carcinoma receiving transcatheter arterial chemoembolization and inhibits cancer cell migration *in vitro*

**DOI:** 10.3389/fonc.2022.1023801

**Published:** 2022-11-11

**Authors:** Yuan Guo, Hong Tao Hu, Shi Jun Xu, Wei Li Xia, Yan Zhao, Xiao Hui Zhao, Wen Bo Zhu, Fang Ting Li, Hai Liang Li

**Affiliations:** ^1^ Department of Minimal Invasive Intervention, The Affiliated Cancer Hospital of Zhengzhou University, Henan Cancer Hospital, Zhengzhou, China; ^2^ Department of Radiology, The Affiliated Cancer Hospital of Zhengzhou University, Henan Cancer Hospital, Zhengzhou, China

**Keywords:** proteoglycan 4, hepatocellular carcinoma, transcatheter arterial chemoembolization, starvation, epithelial–mesenchymal transition

## Abstract

**Purpose:**

To search for adaptive response molecules that affect the efficacy of transcatheter arterial chemoembolization (TACE), analyze their clinical correlation with and prognostic value for hepatocellular carcinoma (HCC), and explore their impact on cell biological behavior and their mechanisms of action.

**Methods:**

HCC tissue gene sequencing was used to identify differentially expressed genes. The expression of proteoglycan 4 (PRG4) in the serum of 117 patients with HCC who received TACE was detected by enzyme-linked immunosorbent assay. Serum-free medium mimicked TACE-induced nutrient deprivation. Cells with stable knockdown of PRG4 (shPRG4) were constructed to verify the effect and mechanism of PRG4 on the biological behavior of HCC cells *in vitro*.

**Results:**

The expression of PRG4 was significantly elevated under TACE-induced starvation conditions. Low PRG4 expression was associated with worse response to TACE treatment, shorter survival time, and stronger HCC migration ability. Furthermore, *in vitro* experiments showed that knockdown of PRG4 promoted HCC cell migration by enhancing epithelial–mesenchymal transition (EMT) while did not affect proliferation. When PRG4 expression was low, starvation treatment impaired the migratory ability of HCC cells and reduced the chemosensitivity of HCC cells to epirubicin.

**Conclusions:**

PRG4 expression predicts survival and TACE treatment response in patients with HCC. Furthermore, knockdown of PRG4 enhanced EMT, leading to HCC cell migration. PRG4 may serve as a biomarker for HCC patients receiving TACE.

## Introduction

Liver cancer is the sixth most common cancer worldwide and the third leading cause of cancer-related death, among which hepatocellular carcinoma (HCC) accounts for the largest proportion (1). Most patients with liver cancer are diagnosed at an advanced stage and lose the chance of surgical resection. The circumstance is associated with the development of occult disease and poor diagnostic techniques (2). In this case, transcatheter arterial chemoembolization (TACE), as a local treatment, is widely used in the treatment of intermediate and advanced liver cancer owing to its minimal trauma and mild side effects and because it can be performed repeatedly, alone or in combination with other treatments (3). TACE kills cancer cells primarily by blocking the arteries that supply blood to the tumor and infusing chemotherapy drugs into the tumor’s interior (4). Cancer cells are in a state of nutrient deprivation (5) and hypoxia (6) after TACE. However, studies have shown that the median survival time of some early patients after liver transplantation or surgical treatment is longer than that of TACE (7). Other cancer therapies, such as Traditional Chinese medicine, have promising effects in preventing metastasis of some advanced HCC as well as drug resistance rather than TACE alone (8, 9). In a word, TACE can significantly improve the quality of life in patients with HCC and prolong their survival though the efficacy of TACE varies greatly among patients. Therefore, there is an urgent need to develop effective biomarkers to guide clinical work.

Proteoglycan 4 (PRG4), also known as lubricin, was originally discovered in the synovial fluid (10) and has been shown to have diverse biological functions, including joint lubrication, anti-adhesion, anti-inflammation, cytoprotection, and matrix binding. Meanwhile, it also contributes to lipid and transforming growth factor beta (TGF-β) retention (11–13). It is upregulated in calcified atherosclerotic plaques and is associated with various joint diseases and dry eye (14, 15).

PRG4 plays an important role in the development of various cancers. High PRG4 mRNA levels are associated with improved survival in patients with HCC, and recombinant human PRG4 (rhPRG4) impairs HCC cell migration. Treatment of CD44-expressing HCC cells with rhPRG4 enhanced the cytotoxicity of sorafenib and regorafenib on cancer cells (16). rhPRG4 inhibits tgfβ-induced invasion of breast cancer cells by inhibiting the downstream hyaluronic acid (HA)-cell surface cluster of differentiation 44 (CD44) signaling axis (17). However, PRG4 also maintains the growth of human myxoid liposarcoma cells by inhibiting IL-24 expression (18). Thus, the role of PRG4 in various cancers remains complex.

The recurrence and metastasis of HCC after treatment is still a puzzling problem (19). Epidemiology shows that HCC is easy to metastasize to distant places through blood or lymph circulation, and about 90% of liver cancer related deaths can be attributed to metastasis, so the metastasis of liver cancer is closely related to prognosis (20). Epithelial–mesenchymal transition (EMT) promotes epithelial phenotype cells to transform into mesenchymal like cells, and plays an important role in the early stage of tumor cell metastasis. The expression of epithelial phenotype proteins, such as E-cadherin, claudins and α-catenin and mesenchymal phenotype proteins, such as N-cadherin, b-catenin, and vimentin, changed during this process (21).

In this study, we evaluated the relationship between serum PRG4 levels and the clinical characteristics as well as prognosis of patients with HCC undergoing TACE. A serum-free medium model (5) was used to simulate the state of extreme nutrient deprivation within the tumor after TACE. Based on this model, we investigated the effect of PRG4 on HCC cells and conducted a preliminary study on its mechanism.

## Materials and methods

### Patients

According to the clinical characteristics of the patients and the treatment response after receiving TACE, five patients (p1-p5) in the treatment response group (CR+PR) and five patients (p6-p10) in the non-responding group (SD+PD) were selected. HCC tissue samples were collected and sent to BGI for gene sequencing. Genes that were differentially expressed between the two groups were selected after analyzing the sequencing results. Subsequently, 117 patients diagnosed with HCC (through radiology or histology) who underwent TACE at Zhengzhou University Cancer Hospital from January 2018 to December 2021 were prospectively recruited. All patients received TACE as the first-line therapy. Patient information and clinical characteristics were collected, including name; sex; age; etiology; preoperative alpha-fetoprotein (AFP), aspartate aminotransferase (AST), and alanine aminotransferase (ALT) levels; presence of cancer capsule; cirrhosis; liver function; tumor size; tumor number; Barcelona Clinic Liver Cancer (BCLC) stage; vascular invasion; intrahepatic metastasis; and histological differentiation. Regular follow-up by telephone and letter was conducted after TACE. Mean postoperative follow-up was 34.0 months (range 6.0–65.0 months). Overall survival (OS) was calculated from the date of TACE to the date of death or last follow-up. Early morning fasting blood samples were collected from the patients the day before TACE, and 62 patients agreed to the collection of additional blood samples on the second postoperative day. All patients provided written informed consent before being included in the study. This study complied with the ethical guidelines of the World Medical Association Declaration of Helsinki and was approved by the Science and Ethics Committee of the Cancer Hospital affiliated to Zhengzhou University (approval number: 2017003).

The inclusion criteria were as follows: 1) the patient meets the criteria for the diagnosis and treatment of HCC, has at least one measurable liver target lesion, and is not fit for surgery or refuses surgical treatment; 2) the age of the patient is between 18 and 75 years; 3) the liver function of the patient is suitable for TACE treatment (Child–Pugh A or B); and 4) the patient has no history of liver cancer-related treatment. The exclusion criteria were as follows: 1) the patient underwent antitumor therapy such as surgery, ablation, or radiotherapy; 2) the patient has severe concomitant diseases, such as severe heart failure or respiratory disease; 3) the patient has uncorrectable abnormal renal function and coagulation function; 4) the patient has severe hepatic dysfunction (Child–Pugh C) or irreversible hepatic decompensation; 5) the patient has an Eastern Cooperative Oncology Group (ECOG) score of >2 points; 6) the patient has insufficient follow-up data; and 7) the patient has a history of other tumors.

HCC was staged according to the BCLC criteria (22). The Child–Pugh score was calculated and measured based on the patient’s clinical tests, laboratory parameters, and imaging findings (23). Response to therapy was assessed 1 month postoperatively using the Modified Response Evaluation Criteria in Solid Tumors (mRECIST) criteria (24) based on enhanced computed tomography or magnetic resonance imaging.

### TACE

All patients were examined by two interventional physicians (HTH and WLX). Epirubicin (Hisun, Zhejiang, China) and lipiodol (Lipiodol Ultrafluide, Laboratoire Guerbet, France) were injected into the blood vessels of the tumor according to the typical TACE protocol, and then gelatin sponge particles (Alicon Pharmaceutical Sci & Tec Co., Ltd., Hangzhou, China) were injected until the blood flow was largely blocked. The embolization therapy was ended when tumor staining disappeared. Additional treatments were based on the size and number of residual tumors, liver function, and patient performance status.

### Blood sampling and ELISA

The patients’ morning fasting venous blood was collected; the sample tubes were centrifuged at 974 × *g* for 10 min at 4°C, and the plasma was fractionated and stored at -80°C. According to the manufacturer’s instructions (Mlbio, Shanghai, China), the plasma samples were diluted 10-fold, and 50 μL of samples/standards was added to the wells. Then, 100 μL of horseradish peroxidase-labeled detection antibody was added to each well and incubated at 37°C for 60 min, and the waste liquid was discarded. Each well was washed five times with 350 μL of wash solution. Then, 50 μL each of substrates A and B was added, the wells were incubated at 37°C for 15 min in the dark, and then 50 μL of stop solution was added to each well. Absorbance at 450 nm was measured using a Thermo Multiskan plate reader (Thermo Fisher Scientific, China). The lowest and highest detectable levels of this kit were 62.5 ng/mL and 2000 ng/mL, respectively.

### Cell culture and starvation treatment

Hep3B and Huh1 cells (Procell, Wuhan) were cultured in Dulbecco’s Modified Eagle Medium (DMEM) (Gibco, USA) containing 10% fetal bovine serum (FBS; Gibco, USA) in a humidified incubator at 37°C with 5% CO_2_. To simulate TACE-induced nutrient deficiency, cancer cells were cultured in serum-free DMEM, and six observation points were set at 0, 1, 3, 6, 12, and 24 h. The subsequent starvation treatment time was the time point with the greatest change in PRG4 expression among the observed time points.

### shRNA constructs and lentiviral production

Three short hairpin RNA (shRNA) sequences targeting PRG4 were designed and cloned into the pLKO.1 vector. The sequences are as follows: shRNA-PRG4-1#, AACAAACCTGAAGAAACAGCT; shRNA-PRG4-2#, CTCGCAGAATTACTGAAGTTT; shRNA-PRG4-3#, AATGGCAATAGGTAGAGATAC. HEK-293T (Procell, Wuhan) cells were transfected with viral and packaging vectors and cultured for 48 h. The viral particles were collected by filtration using a 0.45 mm sodium acetate syringe filter. The virus particles were combined with 8 mg/mL polybrene (Sigma, USA) to infect cancer cells, and 24 h later, successfully infected cells were selected using medium containing 3 μg/mL puromycin (Solarbio, Beijing).

### RNA extraction and quantitative reverse-transcription PCR (qRT-PCR) analysis

Total RNA was extracted using TRIzol (Invitrogen, USA). According to the manufacturer’s instructions (Accurate Biology, Hunan), 250 ng of total RNA was reverse transcribed into cDNA, and PCR reactions were performed in a 10 μL reaction system. The reaction was as follows: 30 s at 95°C, and then 40 cycles of 5 s at 95°C and 30 s at 60°C. The primers used are as follows: PRG4-F, 5′-AGGCCCCATGTGTTCATGC-3′; PRG4-R, 5′-GCGCAAAGTAGTCAGTCCATCT-3′; GAPDH-F, 5′-CCATGGGGAAGGTGAAGGTC-3′; and GAPDH-R, 5′-GAAGGGGTCATTGATGGCAAC-3′.

### Western blot analysis

Cells were lysed in RIPA lysis buffer (Solarbio, Beijing) and centrifuged to extract total protein. The protein concentration of the samples was determined using the BCA method. Protein samples of the same quality were separated in 8% SDS-PAGE and transferred to PVDF membranes. Membranes were blocked with 2% nonfat dry milk in TBST, incubated with primary antibodies overnight at 4°C, and secondary antibodies were added the next day for 1 h at room temperature. The bands were photographed under appropriate conditions using a luminescent reagent (Solarbio, Beijing). Antibodies to detect PRG4 (ab28484), E-cadherin (ab40772), N-cadherin (ab76011), vimentin (ab92547), and β-actin (ab8226) were purchased from Abcam (Shanghai).

### MTT assay

HCC cells were seeded in 96-well culture plates at a density of 2000 cells/well. After a certain period of incubation, 20 μL of 5 mg/mL MTT (Biosharp, Hefei) was added to each well and incubated at 37°C for 2 h. Formazan crystals were dissolved in 100 μL of DMSO. Absorbance was measured at 490 nm using a Thermo Multiskan plate reader (Thermo Fisher Scientific, China). When measuring the 50% inhibitory concentration (IC50) value, 5000 cells were seeded in each well; epirubicin (Hisun, Zhejiang) was added after overnight incubation; ten different measurement concentrations of 0, 0.1, 0.2, 0.4, 0.8, 1.6, 3.2, 6.4, 12.8, and 25.6 mg/mL were set; and the absorbance was measured after culturing for 48 h. Each independent experiment was repeated at least three times.

### Wound-healing assay

Briefly, 4 × 10^5^ cells were seeded in 6-well plates and cultured until 100% confluence. Using a 20 µL pipette tip, a line wound was scratched. The detached cells were washed away with phosphate buffered saline. Serum-free DMEM was then added to culture the cells. The scratches at 0 and 24 h were observed under a light microscope and photographed. Cell migration was analyzed using ImageJ 1.48 software (National Institutes of Health, Bethesda, MD, USA). The wound healing rate was calculated according to the following formula: wound healing rate= [(0-hour scratch area) - (24-hour scratch area)]/[(0-hour scratch area)] ×100%. Each independent experiment was repeated at least three times.

### Transwell invasion analysis

In the upper chamber with a membrane pore size of 8 µm (Corning, USA), 4 × 10^4^ cells were seeded with 100 µL of serum-free DMEM, whereas 700 µL of DMEM with 10% FBS was added to the lower chamber. After 48 h of culture, cells in the upper chamber were removed with cotton swabs, fixed with methanol for 30 min, and then stained with 0.3% crystal violet for 30 min. Invading cells were counted and photographed under a light microscope. Each independent experiment was repeated at least three times.

### Statistical analysis

Analyses were performed using independent paired t-tests, Kruskal–Wallis H test, and one-way analysis of variance tests as appropriate. The cutoff value of sPRG4 was determined based on the area under the receiver operating characteristic curve and the 95% confidence interval. The gold standard is the treatment response evaluated by imaging. Patients were divided into low or high expression groups according to this cutoff value, and the correlation between clinical characteristics of patients and sPRG4 expression was analyzed. Overall survival (OS) was estimated using the Kaplan–Meier method and compared using the log-rank test. Variables that showed a correlation in the univariate analysis were then included in the multivariate Cox regression analysis to determine the independent contribution of each variable to survival. P-values < 0.05 were considered significant. All data analyses were performed using IBM SPSS version 22.0.

## Results

### Correlation of PRG4 expression with treatment response and clinical features in HCC patients

**Figure 1:**
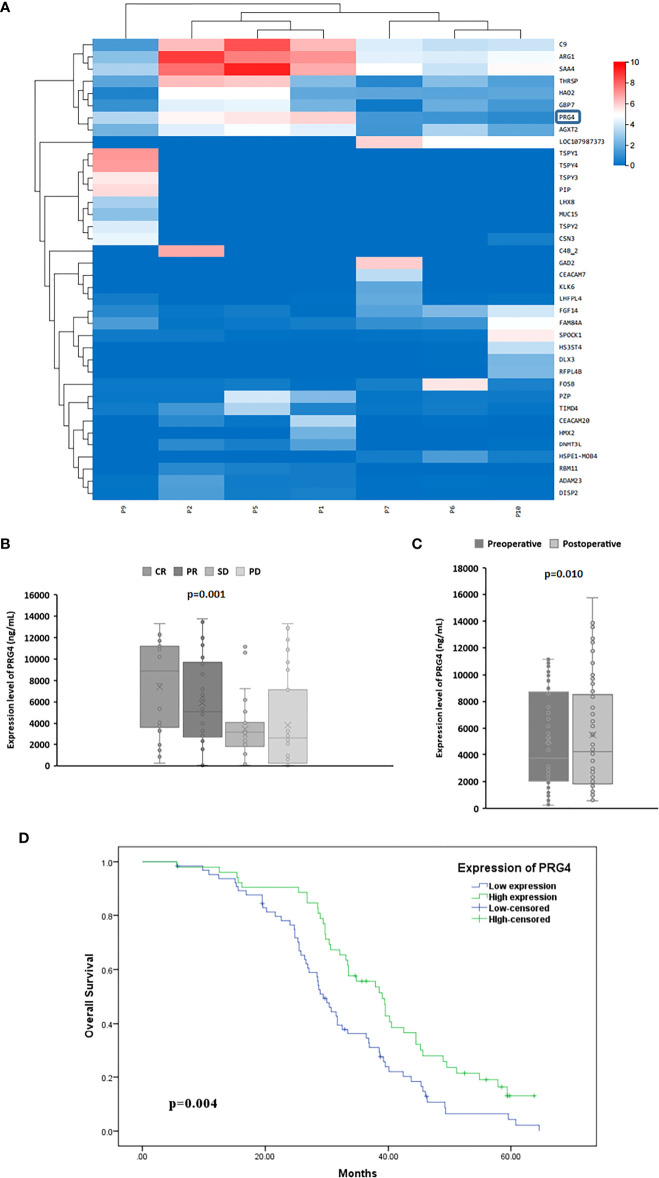
The high expression of PRG4 is related to the better prognosis of TACE. **(A)** Differential clustering heatmap from HCC tissue sequencing. The blue box represents PRG4. **(B)** Serum PRG4 expression levels in patients with HCC who received TACE with different treatment responses. **(C)** Serum PRG4 expression levels preoperatively and on the second postoperative day in 62 patients with HCC who underwent TACE. **(D)** Overall survival of patients with PRG4 high and low expression. CR, complete response; PR, partial response; SD, stable disease; PD, progressive disease.

Since the correlation coefficients of samples p3, p4 and p8 with other samples are all lower than 0.4, that is, their correlation degree is weak or irrelevant, they were excluded from gene expression differential analysis. Combined with the expression level of genes in each sample and the differential expression between the treatment response group (P1, P2, P5) and the non treatment response group(P6, P7, P9, P10), five genes SAA4, ARG1, C9, PRG4 and AGXT2 were selected for ELISA pre-test. The results showed that only the expression level of PRG4 was significantly different between different treatment response groups (p=0.040). Therefore, PRG4 is selected as the research object. Differential expression analysis showed that the expression level of PRG4 in the treatment response group was higher than that in the treatment non-response group (**Figure 1A**). The serum PRG4 expression levels of 117 patients with HCC before TACE were detected by enzyme-linked immunosorbent assay. The expression of PRG4 was significantly different between different treatment response groups (p=0.001, **Figure 1B**). *Post-hoc* pairwise comparison showed that the PRG4 expression level of patients in the CR group (Q50 = 8905.22, Q25–75: 3813.24–11147.91 ng/mL) was significantly higher than that in the SD group (Q50 = 3196.32, Q25–75: 2050.12–3859.23 ng/mL, p=0.017) and PD group (Q50 = 2601.42, Q25–75: 413.21–5249.62 ng/mL, p=0.002). After TACE, the ORR (CR+PR) of high PRG4 group patients was 67.31%, significantly higher than that of low PRG4 group patients (26.15%, p=0.000), which may indicate that high PRG4 expression predicts better treatment response. In 62 patients whose serum PRG4 level was detected on the second day after TACE, the expression level of PRG4 on the second day after TACE (Q50 = 4227.81, Q25–75: 1868.69–8415.58 ng/mL) was significantly higher than that before surgery (Q50 = 3742.39, Q25–75: 2090.15–8618.42 ng/mL, p=0.010, **Figure 1C**).

To investigate the clinical significance of PRG4 in HCC, patients with HCC were divided into low expression (n=65) and high expression (n=52) groups according to a cutoff value (3396.83 ng/mL, AUC=0.823, 95% CI=0.744-0.902). Low levels of PRG4 were associated with higher AFP levels (p=0.044), incomplete capsule (p=0.032), vascular invasion (p=0.014), intrahepatic metastasis (p=0.021), and extrahepatic metastasis (p=0.044), independent of other clinical features (**Table 1**). These results suggest that PRG4 is involved in tumor migration and invasion.

**Table 1:** Correlation analysis between the clinical features and PRG4 expression in HCC.

	Low expression (n=65)	High expression (n=52)	*P* value
Age (≥55/<55)	30/35	23/29	0.836
Sex (F/M)	13/52	9/43	0.711
Etiology			1.000
Hepatitis B virus Hepatitis C virus Others	52 3 10	42 2 8	
AFP level (ng/mL)			**0.044**
≤400 >400	40 25	41 11	
ALT (U/L)			0.377
≤40 >40	29 36	19 33	
AST (U/L)			0.620
≤40 >40	32 33	28 24	
Capsule			**0.032**
Complete Incomplete	21 44	27 25	
Cirrhosis			0.614
Yes No	50 15	42 10	
Child-Pugh class			0.853
A B	57 8	45 7	
Tumor size (cm)			0.724
≤5 >5	43 22	36 16	
Tumor number			0.716
Single Multiple	47 18	36 16	
Vascular invasion			**0.014**
Yes No	32 33	14 38	
Intrahepatic metastasis			**0.021**
Yes No	39 26	20 32	
Extrahepatic metastasis			**0.044**
Yes No	32 33	16 36	
Histological differentiation			0.900
Well Moderate Poor	18 36 11	13 31 8	
BCLC stage			0.215
A B C	21 11 33	22 12 18	

HCC, hepatocellular carcinoma; AFP, alpha fetoprotein; ALT, alanine aminotransferase; AST, aspartate aminotransferase; BCLC: Barcelona Clinic Liver Cancer; The value in bold indicates that the difference is statistically significant (p<0.05).

### Correlation between survival prognosis and PRG4 expression in patients with HCC

The Kaplan–Meier test showed that patients with low PRG4 levels had significantly shorter OS than those with high PRG4 levels (median survival: 29.4 months vs. 39.0 months, p=0.004, **Figure 1D**).

Univariate analysis showed that capsule incompleteness, tumor size, tumor number, vascular invasion, intrahepatic metastasis, BCLC stage, and PRG4 expression were prognostic factors for OS in 117 patients with HCC. Subsequently, the statistically significant variables in the univariate analysis were included in the multivariate Cox proportional hazards regression analysis. Multivariate Cox proportional hazards regression analysis showed that the independent prognostic factors for OS were incomplete capsule (p=0.027), tumor size (p=0.013), tumor number (p=0.015), vascular invasion (p=0.000), and PRG4 expression (p=0.030) (**Table 2**).

**Table 2:** Univariate and multivariate Cox regression analysis of the survival rate of 117 patients with HCC.

Variables	Univariate analysis	Multivariate analysis
	HR (95% CI) *P* value	HR (95% CI) *P* value
Age	0.853 (0.575-1.267) 0.432	
Sex	0.827 (0.504-1.357) 0.453	
Etiology		
HBV HCV	1.219 (0.702-2.118) 0.482 0.867 (0.250-3.009) 0.823	
AFP	0.799 (0.522-1.225) 0.304	
ALT	0.729 (0.486-1.092) 0.125	
AST	1.306 (0.879-1.939) 0.186	
Capsule	1.883 (1.251-2.837) **0.002**	1.721 (1.064-2.782) **0.027**
Cirrhosis	1.175 (0.742-1.861) 0.491	
Child-Pugh class	1.554 (0.807-2.992) 0.187	
Tumor size	2.008 (1.321-3.052) **0.001**	1.767 (1.128-2.768) **0.013**
Tumor number	1.978 (1.287-3.043) **0.002**	1.773 (1.116-2.815) **0.015**
Vascular invasion	3.307 (2.164-5.054) **0.000**	2.861 (1.759-4.656) **0.000**
Intrahepatic metastasis	1.809 (1.211-2.704) **0.004**	1.135 (0.712-1.808) 0.595
Histological differentiation		
Well Moderate	0.685 (0.366-1.284) 0.238 1.129 (0.658-1.938) 0.659	
BCLC stage		
B C	0.666 (0.369-1.205) 0.179 1.772 (1.146-2.740) **0.010**	0.879 (0.466-1.659) 0.691 1.156 (0.696-1.921) 0.576
PRG4	0.562 (0.376-0.840) **0.005**	0.627 (0.411-0.957) **0.030**

HCC, hepatocellular carcinoma; HBV, hepatitis B virus; HCV, hepatitis C virus; AFP, alpha fetoprotein; ALT, alanine aminotransferase; AST, aspartate aminotransferase; BCLC, Barcelona Clinic Liver Cancer. The value in bold indicates that the difference is statistically significant (p<0.05).

These results suggest that PRG4 expression is associated with HCC progression, and low PRG4 expression is associated with poorer prognosis in patients with HCC receiving TACE.

### PRG4 expression in HCC cells is upregulated under starvation

**Figure 2:**
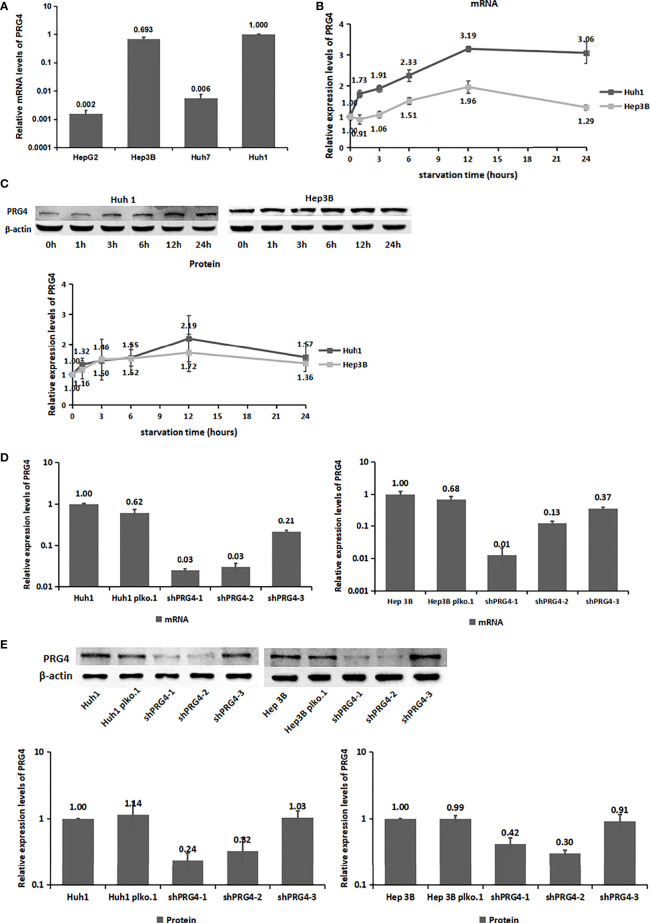
The expression level of PRG4 increased after starvation. **(A)** mRNA expression levels of PRG4 in four HCC cell lines. **(B)** Changes of PRG4 mRNA expression with time of starvation. **(C)** Protein expression level of PRG4 changed with the time of starvation. **(D)** mRNA expression levels were used to detect the knockdown effect of the three sequences on PRG4. **(E)** Protein expression level was used to detect the knockdown effect of the three sequences on PRG4. All data were normalized to control group.

By detecting the mRNA expression levels of PRG4 in four HCC cell lines, HepG2, Hep3B, Huh7, and Huh1, two HCC cell lines with relatively high PRG4 expression, Huh1 and Hep3B, were identified and selected (**Figure 2A**).

Serum PRG4 levels were significantly higher on the second day after TACE than before surgery. To explore the effect of starvation on PRG4 expression, we detected the changes in PRG4 mRNA and protein expression levels in HCC cells after starvation for different periods *in vitro*. Overall, PRG4 was significantly elevated at both mRNA and protein expression levels in Huh1 and Hep3B cells and peaked at 12 h of starvation in the examined time period (0, 1, 3, 6, 12, and 24 h, **Figures 2B,C**). These results suggest that PRG4 is a starvation-responsive molecule under ischemic conditions.

### Knockdown of PRG4 does not affect the proliferation of HCC cells under normal and starvation conditions

As low expression of PRG4 was closely associated with poor prognosis of patients with HCC receiving TACE, we investigated its effect on the biological behavior of HCC cells *in vitro*. Therefore, we constructed cells with stable knockdown of PRG4 (shPRG4) and corresponding negative controls. The knockdown effect of shPRG4-1 and shPRG4-2 was better than that of shPRG4-3 at the mRNA and protein levels (**Figures 2D, E**).

**Figure 3:**
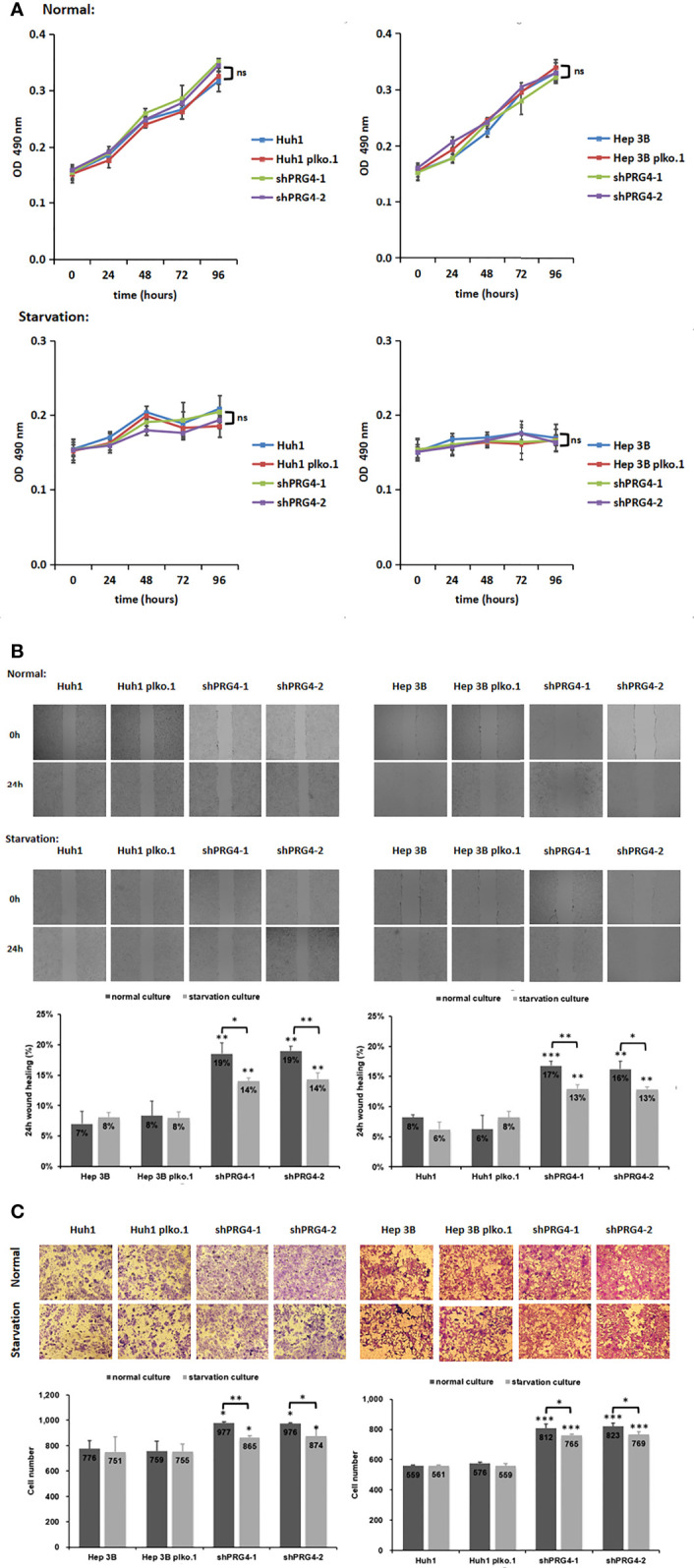
Knocking down PRG4 enhanced the migration of cancer cells but did not affect the proliferation. **(A)** Effect of PRG4 on the growth of HCC cells was detected by MTT assay in normal and starved cultures. **(B)** Wound-healing assays were used to examine the effect of PRG4 on HCC cell migration. **(C)** Transwell assay was used to detect the effect of PRG4 on HCC cell migration. *p < 0.05, **p < 0.01, ***p < 0.001, ns, no significance. All data were normalized to control group.

To explore the correlation between PRG4 and the size of HCC lesions observed in clinical analyses, MTT was used to detect the proliferation of HCC cells. The knockdown of PRG4 did not affect the proliferation of Huh1 and Hep3B cells under normal or starvation conditions compared with that of normal cells and negative controls (**Figure 3A**), indicating that PRG4 does not affect the proliferation and starvation resistance of HCC cells.

### Starvation impairs HCC cell migration enhanced by PRG4 knockdown

To explore the correlation between PRG4 and HCC migration observed in clinical analyses, wound healing assays and Transwell migration assay were used to examine the migratory capacity of HCC cells. Regardless of normal or starvation conditions, the wound healing degree of Huh1 and Hep3B cells after knockdown of PRG4 was significantly higher than that of normal cells and negative controls (**Figure 3B**). At low PRG4 expression, the wound healing degree of starved cultured cells was significantly lower than that of normal cultured cells, and there was no significant difference between normal cells and negative controls.

The same results were confirmed in the Transwell migration assay, as shown in **Figure 3C**. Regardless of normal or starvation conditions, the numbers of Huh1 and Hep3B invading cells after PRG4 knockdown were significantly higher than those of normal cells and negative controls. Only when PRG4 expression was low, the number of invading cells in starved cultures was significantly lower than that in normal cultures. These results suggest that the downregulation of PRG4 promotes the migration of HCC cells, and starvation culture impairs the enhanced migration ability after the downregulation of PRG4.

### Starvation reduces the sensitivity of PRG4-knockdown HCC cells to epirubicin

**Figure 4:**
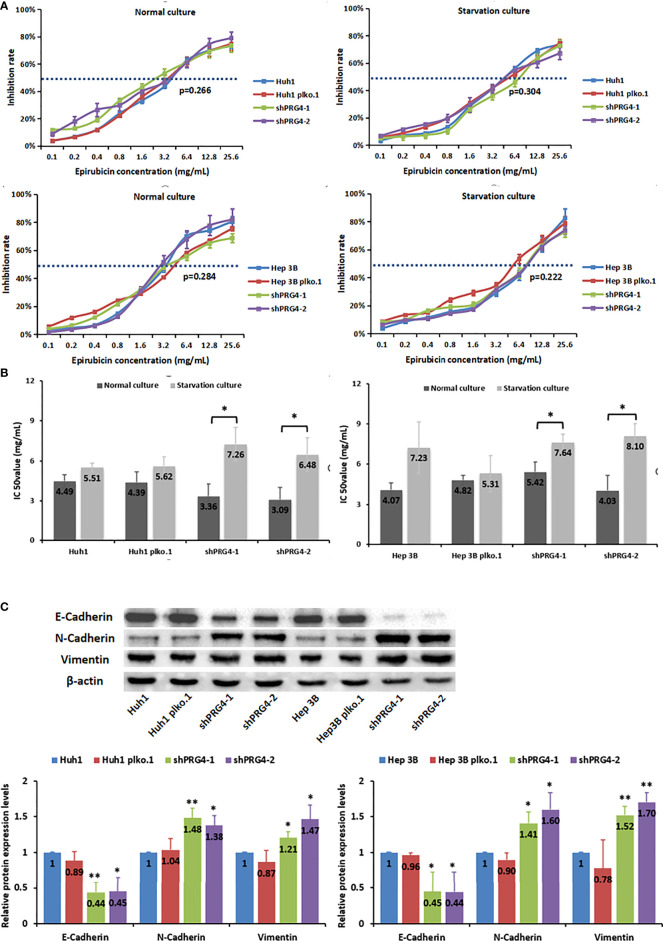
Starvation reduces the sensitivity of PRG4-knockdown HCC cells to epirubicin. **(A, B)** Relationship between the chemosensitivity of HCC cells to epirubicin and the expression level of PRG4 in normal and starved cultures. **(C)** Relationship between PRG4 expression and EMT-related protein expression detected by western blot. *p < 0.05, **p < 0.01. All data were normalized to control group.

To explore the relationship between PRG4 and HCC cell sensitivity to chemotherapeutics in TACE, we measured the IC50 value of epirubicin in inhibiting HCC cells under different culture conditions with different PRG4 expression levels. The expression level of PRG4 did not affect the IC50 values of epirubicin on Huh1 and Hep3B cells whether in normal or starved culture (**Figure 4A**). However, the IC50 value of epirubicin on HCC cells after starvation culture tended to be higher than that in normal culture, but this difference was not significant in normal cells or the negative control group. Conversely, this increase was significant in the PRG4 knockdown group (**Figure 4B**). These results suggest that starvation reduces the sensitivity of HCC cells to chemotherapeutic drugs when PRG4 expression is low but has no significant effect on cells with higher PRG4 expression.

### Knockdown of PRG4 is assocated with epithelial–mesenchymal transition in HCC cells

Comprehensive clinical features and *in vitro* experimental results showed that low expression of PRG4 can promote HCC migration, and it is generally believed that EMT is a key process in the progression of cancer metastasis; thus, we examined whether PRG4 has an effect on EMT. The protein levels of E-cadherin were significantly downregulated, whereas those of N-cadherin and vimentin were significantly upregulated after PRG4 knockdown, compared with those in normal cells and negative controls (**Figure 4C**). These results indicate that EMT in Huh1 and Hep3B cells is enhanced when PRG4 expression is low, which promotes the motility of HCC cells.

## Discussion

According to the BCLC stages of liver cancer, TACE, as a local treatment, can significantly control tumor progression, prolong the OS of patients with intermediate-stage HCC, and can be combined with other treatments to obtain benefits (25, 26). However, poor prognoses, such as recurrence and metastasis after TACE, have long been a concern (27). Therefore, it is necessary to explore key molecules of adaptive responses that influence the efficacy of TACE. We combined the characteristics of TACE to study the role of PRG4 in HCC.

The significant effect of TACE is to block the blood supply arteries of HCC, and the lack of blood perfusion mainly results in a state of hypoxia and nutrient deprivation in the local environment. HCC cells can adaptively induce hypoxia-inducible factor 1α (HIF-1α) under hypoxic conditions after TACE, promoting tumor growth and metastasis and worsening prognosis (28). The nutrient deprivation state caused by TACE can also change the expression of some proteins, such as CD147, which affects the growth of HCC cells. This will also affect further phenotypes and prognosis (5). In our research, the expression of PRG4 in the serum after TACE and transcriptional and translational levels of PRG4 following the starvation culture of HCC cells were increased. Both of which seem to indicate that PRG4 is a protein with increased reactivity after TACE.

Combining the relationship between PRG4 expression and clinical characteristics and prognosis of patients with HCC receiving TACE, it was found that PRG4 seems to affect the prognosis of patients by affecting the metastasis and migration of HCC, which means that the prognosis of HCC patients with high PRG4 expression after TACE is better. This speculation was also confirmed through *in vitro* cell experiments. The migratory ability of HCC cells was weaker when PRG4 expression was high. Interestingly, starvation only impaired the migration of low-expressing PRG4 HCC cells, which may be related to the starvation-induced increase in PRG4 expression.

EMT is widely regarded as a key mechanism of tumor cell invasion and metastasis (29). EMT was promoted with a decrease in E-cadherin expression but an increase in N-cadherin and vimentin expression. Western blot analysis showed that PRG4 may affect the migration ability of liver cancer cells by regulating EMT activity.

Precise local infusion of chemotherapy drugs is also a significant advantage of TACE. Epirubicin is an antibiotic antitumor drug commonly used in TACE to inhibit DNA replication, transcription, and repair by binding to nucleic acids (30). Our study showed that PRG4 did not affect the sensitivity of HCC cells to epirubicin. The effect of starvation, that reduce the sensitivity of cancer cells to epirubicin, was more pronounced when PRG4 expression was low. Starvation can induce autophagy and cycle arrest in cancer cells, and autophagy can reduce their sensitivity to chemotherapeutic drugs (31). This may largely explain why HCC patients with high PRG4 expression have more stable sensitivity to chemotherapeutic drugs and better curative effects after receiving TACE.

This study has some limitations. First of all, although the hypoxic environment caused by TACE does not kill cancer cells as well as nutrient deprivation, more research is needed to determine the significance of hypoxia. Second, because of the large molecular weight of PRG4 and too many repetitive sequences, it is difficult to carry out over expression experiments. In addition, only a preliminary study of the mechanism has been carried out, and a larger sample size is still needed to analyze the specific relationship between PRG4 inhibition of cancer cell migration and the prognosis of HCC patients.

In conclusion, clinical and *in vitro* data suggested that PRG4 expression was upregulated under TACE-induced starvation. PRG4 may impair HCC migration by inhibiting EMT, resulting in better therapeutic response and longer survival in patients receiving TACE. Starvation treatment can weaken the stronger migration ability of HCC cells and reduce the chemosensitivity of HCC cells to epirubicin with low PRG4 expression. PRG4 is expected to be a biomarker for predicting the efficacy of TACE and a potential therapeutic target for the treatment of HCC.

## Data availability statement

The datasets presented in this study can be found in online repositories. The names of the repository/repositories and accession number(s) can be found in the article/supplementary material.

## Ethics statement

The studies involving human participants were reviewed and approved by Science and Ethics Committee of the Cancer Hospital affiliated to Zhengzhou University (approval number:2017003). The patients/participants provided their written informed consent to participate in this study. Written informed consent was obtained from the individual(s) for the publication of any potentially identifiable images or data included in this article.

## Author contributions

Conception and design: HLL, SJX; Patient selection and treatment: HTH, WLX, YG, FTL; Data collection, analysis, and interpretation: YG, XHZ, WBZ, YZ, SJX, HLL; Data interpretation: WLX, XHZ, YZ, HLL; Steering committee activities and critical statistical processing: HLL, HTH; Manuscript writing: YG; Manuscript reviewing: HLL, HTH. All authors contributed to the article and approved the submitted version.

## Funding

This work was supported by the National Natural Science Foundation of China (No. 82002596); the Science and Technology Department of Henan Province (No. 212102310162); and the Medical Science and Technology Research Project of Henan Province (No. LHGJ20190633).

## Acknowledgments

The authors thank Zigang Dong of Zhengzhou University School of Medicine for helpful discussions on topics relevant to this study. The authors would also like to thank Dr. Xilin Feng from Fuheng Biological Technology for constructive criticism and proof-reading of the manuscript.

## Conflict of interest

The authors declare that the research was conducted in the absence of any commercial or financial relationships that could be construed as a potential conflict of interest.

## Publisher’s note

All claims expressed in this article are solely those of the authors and do not necessarily represent those of their affiliated organizations, or those of the publisher, the editors and the reviewers. Any product that may be evaluated in this article, or claim that may be made by its manufacturer, is not guaranteed or endorsed by the publisher.
